# Children’s Perceptions of Dental Experiences and Ways to Improve Them

**DOI:** 10.3390/children9111657

**Published:** 2022-10-29

**Authors:** Melika Modabber, Karen M. Campbell, C. Meghan McMurtry, Anna Taddio, Laura J. Dempster

**Affiliations:** 1 Faculty of Dentistry, University of Toronto, Toronto, ON M5G 1X3, Canada; 2 Department of Psychology, The University of Guelph, Guelph, ON N1G 2W1, Canada; 3 McMaster Children’s Hospital, Hamilton, ON L8N 3Z5, Canada; 4 Leslie Dan Faculty of Pharmacy, University of Toronto, Toronto, ON M5S 3M2, Canada; 5 The Hospital for Sick Children, Toronto, ON M5G 1X8, Canada

**Keywords:** dental fear and anxiety (DFA), fear management, pain management, CARD™ system, dentistry, child

## Abstract

This qualitative study explored children’s perceptions of their dental experiences and their acceptability of the CARD™ (Comfort, Ask, Relax, Distract) system, adapted for the dental setting as a means to mitigate dental fear and anxiety (DFA). A purposive sample of 12 participants (7 males) aged 8-12 years receiving dental care at the Paediatric Dental Clinic, University of Toronto, was recruited. Virtual one-on-one interviews were augmented with visual aids. Participants were oriented to and asked about their perceptions of various dental procedures. Data were deductively analyzed, according to the Person-Centered Care framework (PCC). Four themes were identified: establishing a therapeutic relationship, shared power and responsibility, getting to know the person and empowering the person. Children emphasized the importance of clinic staff attributes and communication skills. They expressed a desire to engage more actively in their own care and highlighted the positive influence of pre-operative education and preparation. Participants found the CARD™ system to facilitate opportunities for self-advocacy in their dental care.

## 1. Introduction

Dental fear and/or anxiety (DFA) is defined as a negative response to real or perceived threat(s) or an apprehension towards a future threat associated with dental treatment or the dental setting [1,2,3,4]. It is the most common psychological condition observed in dentistry, affecting 10–20% of the paediatric population, and includes a variety of physiological, psychological, and behavioral responses [1,2,4,5]. Studies report mixed results, but suggest factors such as generalized fear, previous negative dental experiences, time period since last dental visit, and parental anxiety are correlated with DFA in children [6,7,8,9,10,11,12,13]. Some studies have also reported higher levels of DFA in younger children, females, those with shy-timid personality traits, and negative emotionality [1,2,6,7,8,9,13,14,15,16,17]; however, results vary depending on the study demographics and how DFA is measured [1,2,3,11,12]. Paediatric patients with DFA may exhibit avoidance, aggression, and depression [1,3,4,5,7,12,18] and experience increased perception of and a heightened response to pain [12,18,19,20]. Avoidance behaviour can lead to an increase in dental disease and the need for more complex invasive dental treatments [4,12,18]. Thus, if not managed appropriately, children with DFA may become part of a cycle whereby they avoid or delay seeking dental care, leading to untreated dental disease and more extensive treatment needs, which can generate more DFA [21]. This, in turn, leads to adverse long-term oral health outcomes, including poor oral hygiene, increased caries risk, dental pain, and/or the need for more complicated dental care [12,18,22].

To date, most studies exploring DFA have been quantitative in nature, evaluating children’s experiences through a relatively narrow range of dental scenarios [1,3,4,23,24,25,26]. The limited scope of extant literature is compounded by inconsistency across studies due to variable informants (parents, children) and cut-off scores for evaluating children’s DFA [1,3,11,25,26]. Additionally, some of these measures are outdated and thus of limited relevance in today’s current clinical practices as well as societal norms [23]. In an effort ot address these limitations, qualitative approaches have been used to explore the complex, subjective, and multidimensional nature of DFA in children [23,26,27,28,29,30]. Qualitative studies utilize in-depth interviews to gain perspective on the child’s experiences through their own words [31]. Specific aspects of DFA assessed in this manner include unhelpful thoughts of catastrophizing, emotive distress, anxiety, and heightened autonomic arousal in response to external stimuli (provider attitude, setting design, etc.) [23,27,28,29,30,31]. Qualitative studies highlight the role of social interactions and the child’s level of cognitive development in influencing the degree of DFA. However, to date, most qualitative studies have been conducted in group settings and or with parents present [23,29]. Evaluation of children’s self-reported perceptions of their dental experiences, independent of others, is warranted. 

Beyond an improved understanding of DFA within the paediatric population, there is limited consensus on how to effectively manage it [4,18,32,33]. The CARD™ (C-Comfort, A-Ask, R-Relax, D-Distract) system is a person-centered clinical practice framework that was recently developed to help children cope with vaccine injections [34,35,36,37,38]. Each letter of the word represents a series of interventions aimed to mitigate immunization stress-related responses (ISRR), which include fear, pain, and fainting. When implemented across vaccination settings, CARD^TM^ has reduced ISRR and improved the vaccination experience for both the children and the providers [35,36,37]. The CARD™ system encourages children to actively participate in the delivery of their own care by pre-emptively educating them on various evidence-based coping strategies (in each letter category) and then inviting them to select their desired coping interventions from the letter categories during vaccination [34,35,36,37,38]. Due to the generally transferable principles of this framework, its utility and acceptability within the paediatric dental setting warrants investigation. 

This study sought to qualitatively explore (1) children’s self-reported perceptions of DFA across a wide variety of dental procedures, and (2) the acceptability of the CARD^TM^ system as a dental care delivery framework to help mitigate DFA. 

## 2. Materials and Methods

A qualitative descriptive research design was utilized. Reporting follows the COREQ checklist (https://www.equator-network.org/reporting-guidelines/coreq/, accessed on 27 October 2022).

### 2.1. Study Participants

A purposive sample of healthy (ASA I, II) participants, aged 8–12 years, from the Paediatric Dental Clinic (PDC) at the University of Toronto’s Faculty of Dentistry (Toronto, ON, Canada) was recruited. The relatively narrow age range of children was selected to ensure a sufficient level of developmental and cognitive maturity to comprehend and communicate about the subject matter, while minimizing age-related differences in their developmental maturity [3,39].

Demographic characteristics such as sex, age, ethnicity, and socioeconomic status were considered during participant selection. To minimize recall bias, participants were required to have had at least one dental treatment at the clinic within the preceding 12 months, with treatments limited to non-emergent invasive or non-invasive dental treatments, with or without administration of inhaled nitrous oxide. Due to the COVID-19 pandemic restrictions, one-on-one interviews were conducted virtually via an online virtual video platform (Zoom™). Children with intellectual disabilities, communication difficulties, language barriers, and lack of parental consent for video interviews were excluded.

### 2.2. Study Parameters

Ethical approval for this study was granted by the University of Toronto Research Ethics Board (Protocol #00039905). All participants signed an assent form, and parents signed a consent form. 

Screening of eligible participants was performed through an initial clinic chart review. Parents of eligible children were contacted by a trained clinical coordinator, who provided an overview of the study and ascertained their interest in participating. Parents who expressed an interest were asked for permission to share their contact information with the lead researcher. Those that provided consent were then contacted by the lead researcher (MM) to discuss the study details and to complete the assent/consent process. 

**Figure 1:**
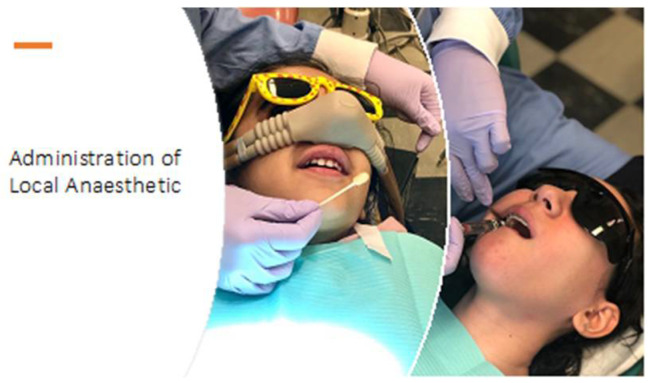
Example of an image shared with participants during the interview.

All participants completed a virtual 1 h one-on-one interview, facilitated by the lead researcher (MM), using a semi-structured interview guide. Participants were specifically asked about their perceptions about aspects of their dental appointments, including: (1) waiting area, (2) dental chair, (3) dental exam, (4) dental suction, (5) dental Xray imaging, (6) dental cleaning, and (7) invasive procedures involving nitrous oxide sedation and/or local anesthesia administration, and (8) fillings involving use of drill. The guide included prompts to remind children of previously encountered procedures with accompanying images shared via the computer (see examples in Figure 1 and Table 1). Participants were then introduced to CARD™ and coping options in the different letter categories adapted to the dental context (Table 2). They were asked to provide their opinions about CARD™, and to select coping strategies they would want to use. All interviews were audio-recorded and transcribed verbatim. Parents may have been present (and assisted children with technical issues), however, they did not answer any questions during the interview.

The lead researcher (MM) is a dentist who at the time of the research, was undertaking a masters degree in pediatric dentistry. She has experience providing dental care to children and is interested in providing high quality care that minimizes stress for children and their families. She was not directly involved in the delivery of dental care of any of the participants. Other research team members, also not involved in the care of participants, included a dentist (KMC), a dental hygienist (LJD), a clinical psychologist (CMM) and a pharmacist (AT). The study team was knowledgeable in the relevant content areas and qualitative methodologies. 

The interview guide was first pilot tested on two children (6 and 8 years old) and modified by the research team to improve clarity before formal recruitment for the study. The data from the first two interviews were not included in the analysis. There were no field notes and no participant was re-interviewed. No participants reviewed their transcripts for accuracy or provided feedback on the findings as part of their participation. 

### 2.3. Sample Size

Sampling size was determined with the goal of capturing the complexity and range of participants’ experiences to enable meaningful exploration. Moreover, the concept of data saturation, where no new further substantive information would be obtained from further participants was considered [23,40,41,42,43]. Based on prior studies using this interview format, it was anticipated that 12–15 interviews would be necessary to reach data saturation [23,43]. 

### 2.4. Conceptual Frameworks and Data Analysis

The research paradigm was post-positivist. Deductive content analysis was used to analyze the data using an adapted version of the Person-Centered Care (PCC) framework [44,45,46]. The PCC framework identifies six main domains: (1) Establishing a Therapeutic Relationship; (2) Shared Power and Responsibility; (3) Getting to Know the Person; (4) Empowering the Person; (5) Trust and Respect; and (6) Communication [44]. Interviews were transcribed verbatim, reviewed and coded using a modified version of this framework, which only included the first four PCC domains to minimize repetition and overlap [44]; each theme contains subthemes which were derived from the study. Data from the transcripts were analyzed by the lead researcher (MM) using NVivo12 software (QSR International, LLC). Recurring meetings were held with the senior authors (LD, AT, CMM) to discuss the logic, consistency and trustworthiness of the subthemes and themes as applied to the data, until consensus was reached. The decision to stop the study was determined based on review of the results and lack of new themes.

## 3. Results

Overall, 50 eligible children were approached for participation and 12 participated. Fourteen families did not respond to invitations, 13 refused to participate, 7 did not return the assent/consent forms, and 4 did not meet eligibility criteria. Interviews were conducted from October 2020 to November 2021. Data saturation was reached with 12 interviews. 

### 3.1. Description of Participant Characteristics

The characteristics of the participants are summarized in Table 3. They were diverse in terms of age, gender, ethnicity, and dental experience.

### 3.2. Description of Themes, Subthemes, and Associated Sample Participant Quotes

Children’s dental experiences were categorized within four major themes with associated subthemes (summarized in Table 4). An explanation of the themes with sample participant quotes, is provided below, whereby participants are identified as P1 (Participant 1), P2 (Participant 2), etc.

#### 3.2.1. Theme 1: Establishing a Therapeutic Relationship

This theme defines provider characteristics that help establish a meaningful relationship between the participant and the provider. 

##### Dental Team Characteristics

Children identified that providers who demonstrated warmth and kindness, and those who took a keen interest in them helped promote trust and enhanced their care. For example,

*“They comforted me. They let me squeeze their hands. I was really scared. They helped me get through it. They were kind.”*

P3

Conversely, when children perceived the professional team to be disinterested in them as individuals, they felt dissatisfaction towards their care and a dampened patient-provider relationship.

*“They ask basic questions every time I go there. The same questions over and over again. It gets boring. I wish they would ask me personal questions and tell me about themselves.”*

P2

##### Effective Open Communication

Participants placed great emphasis on verbal and non-verbal communication in establishing a positive patient-provider relationship. Effective verbal communication included active listening, the use of age-appropriate explanations and honest bidirectional exchange of information. The use of supportive non-verbal gestures, such as a pat on the back, a nod, or holding of hands, particularly during DFA-provoking aspects of the dental treatments were also reported to be important. The combined use of effective verbal and non-verbal cues helped gain trust and a sense of security amongst the children as described by P11 below.

*“It made me feel nervous at first. But [the dentist] always listened to what you were saying, and they never ignored you. They kept the conversations going. I felt happy because there were finally some people who understood what I was talking about, and they really listened to me.”*

P11

#### 3.2.2. Theme 2: Shared Power and Responsibility

This theme describes children’s desire to actively engage in their clinical care and feel a sense of ownership in the care provided. 

##### Personal Autonomy and Advocacy 

Children expressed a desire for autonomy and opportunities for self-advocacy during dental encounters. Such opportunities provided them with a higher degree of control and ownership over their care, which enhanced their overall experience. For example,

*“I would like to pick the flavours of polishing paste/topical fluoride. It would make my mouth more comfortable since I cannot eat anything.”*

P2

The ability to verbalize treatment needs and have their feedback acknowledged and incorporated into their care made the children feel included in the decision-making process, as elaborated by P4.

*“Sometimes using the suction makes your mouth really dry. Closing your mouth is helpful, but most times it is not possible because your mouth must be open, making your mouth dry. A few times I asked to get it removed. [When the dentist removed it], it was better because my mouth felt normal again.”*

P4

##### Self-Directed Support

Children discussed the role of self-coping techniques when undergoing a DFA-provoking treatment (e.g., receiving a local anaesthetic injection). The use of deep breathing and positive self-talk was used by some participants to divert attention away from stressful stimuli and to help them to relax. This in turn enabled them to reframe their negative emotional state into a more positive experience.

*“By breathing it takes my mind off of some of the stuff that hurts, and I feel better.”*

P8

Children also described the use of positive self-talk and daydreaming to be important in helping to complete the treatment. Older children used self-talk techniques to help internally rationalize the need for the proposed treatment and accept the discomfort they experienced, as described by P11 (10 years old) below.

*“It’s just part of life [dealing with unpleasant experiences]. You are going to have to deal with it. It doesn’t matter if it hurts. So, before [the treatment] I get my body prepared. [This way] I can be calm, and focus. That will help me get ready for it.”*

P11

#### 3.2.3. Theme 3: Getting to Know the Person

Within this theme, participants highlighted the importance of the provider learning about their experiences and wishes to help improve their overall dental experience. 

##### Participants’ Lived Experiences

Children’s past dental experiences, shaped by both extrinsic (e.g., dental team characteristics, procedural elements, dental setting) and intrinsic (e.g., participant age, pain and fear experiences) factors, influenced their perception of future dental encounters. Children wanted their provider to know and understand these factors and implement appropriate modifications during the dental appointment to improve their experience. Regarding extrinsic aspects, the participants felt that the clinic setting/environment was overwhelming and unappealing. Specifically, they expressed dissatisfaction with the colour scheme of the waiting area and clinical rooms, as well as the busy waiting area, which lacked age-appropriate activities as stated by P12.

*“There are lots of chairs next to each other and there is nothing to do there. You are just thinking and waiting for your appointment.”*

P12

Procedural elements were also described as painful and anxiety provoking. Participants expressed fear about the sight of the needle and the auditory/tactile sensations of the tools, for example:

*“The drill is the scariest part because it’s very sharp and has a loud sound.”*

P4

Children also reacted negatively to the scent and bitter taste of the various dental materials, particularly the anaesthetics (topical, local).

*“The medicine from the needle was really uncomfortable. I felt weird stuff coming into my mouth and it just tasted bitter.”*

P5

Intrinsic factors, such as catastrophizing and negative internal thoughts also influenced children’s perceived experiences. Younger participants reported increased vulnerability to negative internal thoughts. They were worried about being harmed (e.g., injured, suffocation) during their dental appointment.

*“I think the X-ray machine would bump into me, and I would fall, hit my head or get hurt.”*

P7

In some children, their expressed DFA was also a manifestation of a conditioned response from previous negative health-related encounters. These children expressed fear of reliving past negative experiences, whether directly encountered or observed in others.

*“Well, with the needles I was really scared. I am afraid of getting the flu shot, and it reminded me of that. So, I didn’t want to do it.”*

P3

Children also perceived negative social judgment from other children in the clinic, and thus were less likely to express their emotions fully and sought increased privacy, as shared by P3.

*“Some people get scared [during their dental visits], and they cannot let their feelings out because people are looking at them. Instead of fearing everything they can have privacy.”*

P3

##### Participants’ Wishes, Needs, and Values

Children described their preferences for the dental environment, pain management, information provision (i.e., preparation/education), and family involvement. Firstly, ideal dental settings incorporated bright colours, synchronous design, and access to child-friendly play areas with audiovisual tools for distraction within the waiting room.

*“We could set up a few drawing activities in the corner, this way the children would have something to do instead of just being bored of waiting.”*

P4

Secondly, they wanted procedures to be short (<45 min) and pain free. For example, as reported by P5, children expressed a desire to use sedation (nitrous oxide sedation) in most visits, particularly those considered to be invasive.

*“If there’s more laughing gas and we could use laughing gas a little bit more often for the freezing.”*

P5

Provider-led distractions (telling jokes, songs, stories) were desired by most children, as it directed their attention away from unpleasant stimuli. Distractions were seen as most critical for invasive procedures.

*“…Maybe not having them see the needle or having them distracted is much more helpful. You could have them [the dentist] talk about something else so that they don’t have to worry about the needle at all.”*

P4

Children also expressed interest in smaller or cushioned X-ray films to mitigate pain and the risk of soft tissue trauma. For example,

*“The paper film cuts my mouth and is close to my throat. It feels like I am going to swallow it. Maybe they can put a clip or something soft on it.”*

P2

Thirdly, the scope and extent of information sought by the children about their care varied. Most participants described seeking some information about disease processes, the rationale for treatment, and procedural steps.

*“The dentist can explain steps and show the materials that they have so you know they don’t hurt. That way you are not nervous and know you will be okay.”*

P12

Participants advocated for a pre-emptive warning of possibly painful stimuli to help them mentally prepare for it. This would also enable them to seek more information if they wished. Children also described the need for demonstration and modeling of tools prior to procedures. Demonstration of how tools work prior to their use helped familiarize children with them and procedural steps, as well as potential sensory perceptions. This alleviated fear associated with not knowing what would happen and preventing negative predictions, as explained by P8:

*“Maybe the dentists can show me the tools that they are using. Maybe I can hold some of the instruments that way I would not fear them. It would help if they showed me the tools and let me hold them.”*

P8

Fourthly, a crucial factor in optimizing participants’ dental experiences was parent/caregiver involvement. Parental presence, particularly that of the mother, was seen as particularly helpful during the initial visit or during an invasive procedure (e.g., administration of local anaesthetic) because they could provide security and comfort.

*“Having my parents there would make me feel safe. When they are there, nothing would happen to me.”*

P9

Parental presence also increased the perceived sense of control during times of vulnerability during procedures. For younger children or those who described themselves as “shy”, parental presence mitigated separation anxiety and fear of treatment.

*“I am used to having my mom stay with me. I get really nervous if I am around people and my mom isn’t there. Having her there makes me comfortable because I am used to seeing her.”*

P3

#### 3.2.4. Theme 4: Empowering the Person

Empowerment entails the elements and resources desired by the individual to successfully navigate the dental visit and associated procedures. Learning about the CARD™ system and choosing coping interventions was perceived to increase the sense of empowerment.

##### The CARD™ System

Participants felt that the CARD™ framework could play an important role in facilitating opportunities for self-advocacy. They perceived that CARD™ would enable them to voice their preferred coping strategies at various stages of the dental encounter.

*“CARD™ is really good. I [get] to choose my own favourite cards and choose when to use it on my own and where I would do it.”*

P10

Participants highlighted the utility of CARD™ for invasive procedures, such as the administration of local anaesthetic. For such DFA-provoking moments, CARD™ would allow children to advocate for preferred coping strategies and enhance their sense of control.

*“The CARD™ system was very great. You can have Ask, Comfort, Relax, and Distract, they are very good if you’re very scared, stressed, or tired. It’s a very good improvement for the dentist.”*

P12

##### Coping Strategies

Children believed that interventions consistent with the CARD™ system would improve their dental experience. Children with DFA towards a specific tool or procedure (e.g., dental suction or dental imaging) preferred to engage in strategies that integrated information provision and demonstration of the tools and procedures prior to use, consistent with the “Ask” category of CARD™.

*“The dental X-ray makes me uncomfortable. I want to ask, ‘Can I see what the dentist is doing?’ so I can know what’s going to happen in advance and have the dentist tell me what actually is going to happen.”*

P2

*“For the dental suction, I would say ‘How will it feel?’ That way I would be prepared for it, so if I had the dental suctioning I would not freak out.”*

P11

For these participants, “Ask”-based strategies provided opportunities for disclosure of information deemed necessary to familiarize them to the tool, enhanced treatment predictability, and provided mental readiness to face their fear.

Conversely, those who reported more generalized DFA or catastrophizing preferred coaching by the provider, Comfort (e.g., favourite object), and Relaxation (e.g., breathing techniques).

*“My favourite card would behaving a favourite object or toy like a stress ball or squishy, just because when you are really stressed, having that object in your hand and squishing it diverts your mind off of it.”*

P4

*“I like to take deep belly breaths. some deep breaths can help you calm yourself down if you are stressed or scared to go [see the dentist]. You can take deep breaths that way you know you are going to be okay and that everything is going to be fine.”*

P12

All participants perceived parental presence to be of utmost importance to the success of their dental encounter.

*“I want my parents to sit next to me. That way I would not be scared,because my mom makes me comfortable and I know if I feel something wrong, my parents will help me.”*

P8

Further, children hypothesized better treatment outcomes through the incorporation of (i) distraction techniques (e.g., toys), (ii) nitrous oxide sedation, (iii) treatment breaks, and (iv) privacy. Each of these will be explored below.

##### (i) Distraction

Distraction was believed to be helpful in the waiting area to lessen boredom and help pass the time. For some, distractive tools were also effective in helping reduce experienced anxiety while waiting for their treatment in the waiting area.

*“I would like towatch a movie or have a video to distract me. That way I would be distracted from anything I’m worrying about if I’m sitting on a chair waiting for what is going to happen.”*

P11

During invasive procedures, these forms of distraction would help divert their attention away from the anxiety provoking dental instruments, as explained by P10:

*“I can listen to music. I can concentrate on the music and not think about what would happen and feel calm.”*

P10

##### (ii) Nitrous Oxide Sedation

During anxiety-provoking procedures, participants identified sedation via nitrous oxide to significantly mitigate their needle-related pain.

*“I want the laughing gas. I think it helped a lot. When they gave me the needle, I felt the pain. But with the laughing gas I didn’t feel anything. It was a lot more comfortable than the needle.”*

P5

##### (iii) Treatment Breaks

The opportunity for short breaks was highlighted as important. Following the initial provision of information, rest breaks allowed children to review and process the information, gather their thoughts, and plan coping strategies.

*“Before treatment, I want a break so I can think about things, take a deep breath so that you are ready if something bad is about to happen.”*

P6

Breaks were also highly valued during procedures, allowing for a brief respite during unpleasant aspects of the dental treatment. The incorporation of such breaks also helped minimize muscle fatigue from prolonged jaw opening, as stated by P4:

*“I want to ask to take breaks. Sometimes it gets really tiring to stay [with my mouth] open in one position for a long time.”*

P4

##### (iv) Privacy

Children expressed interest in having privacy during DFA-provoking procedures which would allow them opportunities to truly express their emotions without fear of judgment or embarrassment.

*“It would be the privacy; you don’t want other people looking at you or worrying about you. So, I prefer privacy.”*

P3

## 4. Discussion

This study aimed to explore children’s dental experiences and the acceptability of the CARD™ system, modified for the dental setting. Children’s dental experiences were categorized within four of the PCC domains: (1) establishing a therapeutic relationship, (2) shared power and responsibility, (3) getting to know the person, and (4) empowering the person. Children emphasized the establishment of a therapeutic patient-provider relationship. This relationship was influenced by provider characteristics of empathy and kindness, understanding the child’s needs, and by bidirectional open communication. Shared power and responsibility reflected the need for children to understand the procedure, express their wishes, and take ownership over certain aspects of their dental care. Children expressed that providers need to understand the children and facilitate their participation. Children were highly receptive to incorporating CARD™ in their future dental encounters as they believed it would improve their experience through empowerment and self-advocacy. Within this framework, coping strategies that were highlighted for the dental context included distractive items, administration of nitrous oxide sedation, treatment breaks, and privacy. 

The findings of this study are in keeping with prior literature highlighting the impact of clinician attributes in mitigating DFA [3,4,12,18,23,47,48]. Expressions of empathy and effective communication using a calm, non-judgmental tone, which facilitates an open bidirectional dialogue, has been reported to help mitigate DFA, improve the child’s dental experience, and result in better treatment outcomes [4,12,18,23,29,48]. These findings suggest that communication is integral to the formation of trust and rapport between patient and provider that helps mitigate DFA in children [11,12,18]. 

Consistent with extant literature, our study identified the importance of child-friendly settings, processes, and procedural pain management to minimize DFA. Morgan et al. (2017) observed that calm and caring providers, complemented with a non-threatening and child-friendly atmosphere led to increased trust and comfort by both patients and their parents [23]. The use of soft music, dim lights, and pleasant ambient odour in the dental environment induced an anxiolytic effect upon children [4]. Conversely, prolonged waiting times, loud sounds produced from instruments, medicinal scents and the lack of engaging and age-appropriate distractive tools or activities perpetuated negative thoughts and higher rates of DFA [4,12].

Our results are consistent with our work in the vaccination context as well, whereby children identified knowledge and health provider support for their preferred coping strategies as important to improving their experiences [34,37]. Children found the CARD™ system to be a useful educational framework to learn about dental care, enable self-advocacy for their preferred coping strategies, and to help reduce DFA. While use of the CARD^TM^ framework has led to lower rates of ISRR (including fear, pain, and dizziness) during vaccinations, this is the first study to examine its acceptability in the dental context. Study participants expressed a strong desire for incorporating the CARD™ system during future invasive dental procedures. The preferred coping interventions within the CARD^TM^ framework varied based on the type of proposed treatment and the level of DFA experienced. This reiterates the need for an individualized approach to mitigating DFA within the dental setting. Children expressing fear towards specific stimuli desired information provision with gradual stimulus exposure. On the other hand, those with more generalized fear preferred emotional support (parental presence), distractive strategies, and relaxation strategies (deep belly breathing). This is similar to the findings of others, whereby in children with specific fears, systemic desensitization through graded exposure were found to be most effective in mitigating fear [18,22], and in children experiencing general fear, relaxation strategies (e.g., breathing), emotional support, reassurance, distraction and pharmacological interventions (nitrous oxide sedation) have been most impactful [22,23,29]. These findings serve to demonstrate the potential of a multifaceted intervention approach such as CARD™ in managing DFA. 

### Strengths and Limitations

The present study is one of the few studies to directly engage children in assessing their dental experience and the factors which contribute to DFA. This approach enabled a clearer understanding of children’s lived experiences, values and preferences, without the influence of secondary observers, such as parents, other children or dental care providers. This is also the first study to assess the acceptability of the CARD™ framework within the dental context. This study provides the groundwork for future research to evaluate the effectiveness of CARD™ to mitigate DFA and promote an optimal dental experience for children. 

Limitations of our study include the potential for recall bias since the interviews were not conducted in the dental clinic in real-time. However, we did present images of the specific clinic setting and explained procedural steps to participants as memory prompts; participants appeared to remember and understand as exhibited by their responses. Nevertheless, their recollections may have been impacted by participation after the event. All participants were English-speaking and willing to discuss their dental experiences. While our analysis suggests that saturation was achieved, the views and experiences of individuals not represented within our study sample may not have been adequately captured. For instance, our study may have attracted children with particular beliefs and attitudes around their dental experiences. Further, the nature of our study (i.e., qualitative vs. quantitative) does not allow us to examine correlations among identified factors, such as gender, age, or ethnicity on dental fear and anxiety. We found no demonstrable difference, however, between male and female children in their narratives. Lastly, the data is subject to social desirability bias as children’s responses may be influenced by their desire to please the researcher. 

## 5. Conclusions

Many factors contribute to DFA within the paediatric population. Children express a desire for their active involvement in their dental care and a more individualized approach to coping strategies to help mitigate their DFA. The CARD™ system is a framework that may help facilitate a more person-centered approach to the delivery of dental care in children and thus a potential avenue to optimize their oral health. Future studies are recommended that further this work, including examining: (1) children’s dental experiences in real time and in different dental settings, (2) acceptability of the CARD™ system to dental staff, and (3) feasibility and impact of the CARD™ system when implemented in the delivery of paediatric dental care.

## Figures and Tables

**Table 1:** Example of information shared with participants and associated questions during the interview.

Dental Procedure and Description	Associated Questions
*Administration of local anaesthetic* Sometimes the dentist puts your tooth/teeth to sleep for a while so they can fix them without bothering you. To do this, they first apply a jelly beside your tooth which numbs the area. Then, they give you a needle.	Do you remember ever having the dentist put your tooth/teeth to sleep? If NO, then move to the next question. If YES, proceed with the questions below: (a) How do you feel when the dentist puts your tooth/teeth to sleep? (Probe based on answer) (b) What are ways that you think we can make putting your tooth/teeth to sleep better and more comfortable for you?

**Table 2:** CARD™ (Comfort Ask Relax Distract) options provided to participants during the interview.

CARD™ Letter Category	Coping Options
Comfort	○ Wear comfortable clothing
	○ Have a parent sit next to you
	○ Have a parent comfort you (hold your hand or rub your shoulder)
	○ Hold a favourite object or toy
	○ Have privacy
Ask	○ Why am I going?
	○ What will happen?
	○ Can I see what the dentist is doing?
	○ How long will it take?
	○ How will it feel?
	○ Can I take breaks?
	○ How will the dentist know when I want to take a break?
	○ Can my parents stay?
	○ Can I bring a favourite object or toy?
	○ Can I wear the ‘astronaut nose’ (laughing gas)? *
	○ What will the ‘astronaut nose’ feel like? *
Relax	○ Take deep belly breaths (pretend you are blowing a balloon)
	○ Do some self-talk (tell yourself you can handle this)
	○ Wear the ‘astronaut nose’ (laughing gas) *
	○ Have a parent stay with you
	○ Have the dentist help you practice relaxation techniques
	○ Tense your muscles to prevent dizziness
Distract	○ Listen to the dentist tell stories, jokes or sing
	○ Listen to a parent tell stories, jokes or sing
	○ Watch a movie or video
	○ Listen to audiobook or music with headphones
	○ Hold your favourite object or toy
	○ Allow yourself to daydream

* only shown to participants that had prior experience with the intervention.

**Table 3:** Summary of participants’ characteristics (*n* = 12).

	Mean (SD) or Frequency (%)
Gender, male	7 (58%)
Age, years	9.8 (1.48)
Ethnicity Asian (South Asian/West Asian) White/European Black/African/Caribbean Mixed Ethnicity	5 (42%) 3 (25%) 2 (17%) 2 (17%)
Total number of visits	8.5 (9.02)
Types of visits Invasive procedures ^1^ Non-invasive procedures ^2^	9 (75%) 3 (25%)

^1^ Invasive procedures are defined as procedures requiring the manipulation of gums, removal of tooth structures, or perforation of oral tissue (e.g., administration of local anaesthetic and restorative treatments such as fillings, with or without administration of nitrous oxide sedation). ^2^ Non-invasive procedures include X-ray imaging, mouth exams, dental cleanings, and fluoride application.

**Table 4:** Summary of themes and subthemes.

Themes	Subthemes and Descriptions
(1) Establishing a Therapeutic Relationship	(a) Dental Team Characteristics Provider attributes could negatively or positively impact on DFA (b) Effective and Open Communication Children preferred types of communication (e.g., honest and open communication)
(2) Shared Power and Responsibility	(a) Personal Autonomy and Advocacy Children expressed desire to verbalize treatment needs and actively participate in treatment decision making (b) Self-Directed Support Children expressed desire to engage in self-coping techniques
(3) Getting to Know the Person	(a) Participants’ Lived Experiences Children expressed their desire for dentists to be cognizant of their feelings about different parts of their appointment (e.g., boredom in the waiting area and DFA associated with invasive procedures) (b) Participants’ Wishes, Needs, and Values Children expressed their preferences for a more ‘child-friendly’ clinic, pain management, information (i.e., preparation/education), and parental presence
(4) Empowering the Person	(a) The CARD™ (Comfort Ask Relax Distract) system Children expressed that CARD™-based interventions provided opportunities for self-empowerment and self-advocacy (b) Coping Strategies Children identified specific coping strategies as important, including distractive strategies, nitrous oxide sedation, breaks, and privacy, particularly during DFA-provoking procedures

## Data Availability

Data are not available.
